# Acupotomy Therapy for Knee Osteoarthritis Pain: Systematic Review and Meta-Analysis

**DOI:** 10.1155/2020/2168283

**Published:** 2020-10-31

**Authors:** Jigao Sun, Yan Zhao, Ruizheng Zhu, Qianglong Chen, Mengge Song, Zhipeng Xue, Rongtian Wang, Weiheng Chen

**Affiliations:** ^1^The Third Affiliated Hospital of Beijing University of Chinese Medicine, Beijing, China; ^2^Wangjing Hospital, China Academy of Chinese Medical Sciences, Beijing, China; ^3^Guizhou University of Traditional Chinese Medicine, Guiyang, Guizhou, China

## Abstract

**Methods:**

We performed a comprehensive search on PubMed, the Cochrane Library, EMBASE, and four Chinese databases for articles published prior to June 2020. We included only randomized controlled trials (RCTs) that used acupotomy therapy as the major intervention in adults with knee OA, were published in either Chinese and English, included more than 20 subjects in each group, and included pain and function in the outcome measures. Knee OA was defined by the American College of Rheumatology or Chinese Orthopedic Association criteria in all studies. We extracted the visual analogue scale (VAS) pain score, the Western Ontario and McMaster Universities Osteoarthritis Index (WOMAC) pain score, the total effectiveness rate, the modified Japanese Orthopedic Association (JOA) activities of daily living score, and Lysholm's score. We calculated the mean difference (MD) or risk ratio (RR) for all relevant outcomes. Meta-analyses were conducted using random-effects models when appropriate.

**Results:**

We identified 1317 potentially relevant studies, thirty-two of which met the eligibility criteria and were conducted in China between 2007 and 2020. A total of 3021 knee OA patients (62.96% female, median age: 57 years, and median disease duration: 33 months) were included. The treatment duration ranged from 1 week to 5 weeks (median: 3 weeks). The typical acupotomy treatment involved releasing soft tissue adhesions and was performed once a week for 1–5 weeks until the pain was relieved. The control group treatments included acupuncture (8 studies), electroacupuncture (10 studies), sodium hyaluronate (8 studies), radiofrequency electrotherapy (1 study), and nonsteroidal anti-inflammatory drugs (NSAIDs, 5 studies). The results from the meta-analysis showed that acupotomy led to superior improvements in the VAS pain score (MD = −1.11; 95% confidence interval (CI), −1.51 to −0.71; *p* < 0.00001) and WOMAC pain score (MD = −2.32; 95% CI, −2.94 to −1.69; *p* < 0.00001), a higher total effectiveness rate (RR = 1.15; 95% CI, 1.09–1.21; *p* < 0.00001), and superior improvements in the JOA score (MD = 6.39; 95% CI, 4.11–9.76; *p* < 0.00001) and Lysholm's score (MD = 12.75; 95% CI, 2.61–22.89; *p* = 0.01) for overall pain and function. No serious adverse events were reported.

**Conclusion:**

Chinese acupotomy therapy may relieve pain and improve function in patients with knee OA. Furthermore, rigorously designed and well-controlled RCTs are warranted.

## 1. Introduction

Symptomatic osteoarthritis (OA) is the most frequent cause of dependency in lower limb tasks among ageing populations and is associated with substantial physical and psychosocial disability, a reduced quality of life, and substantial healthcare costs [1]. At present, knee OA is considered a common health problem worldwide; in the United States, nearly 40% of adults over the age of 60 suffer from this disease [2]. Currently, no effective disease-modifying remedies are available to treat knee OA [3]. In the absence of effective disease-modifying treatments, the current standards of care for knee OA are primarily aimed at pain relief and functional improvement [4].

Nonsteroidal anti-inflammatory drugs (NSAIDs) are important for treatment due to their widely reported efficacy but are restricted in clinical applications due to their side effects [5–9]. Therefore, only topical NSAIDs are strongly recommended for individuals with knee OA in the 2019 OARSI guidelines [10]. Due to the side effects of drugs, complementary and integrative therapies are favoured in the treatment of OA. Many complementary and integrative methods are used in China, such as traditional Chinese medications, and it has been reported that the prevalence of knee OA is 18% in China [11, 12]. In the latest 2019 American College of Rheumatology/Arthritis Foundation guidelines, tai chi, a traditional Chinese exercise, is strongly recommended, and acupuncture is conditionally recommended [13], but there is no mention of acupotomy therapy, which is also an important complementary and integrative therapy.

Acupotomy therapy is widely used in Chinese clinical practice and recommended in the Chinese medicine expert consensus for knee OA [14]. Acupotomy is a type of acupuncture used in traditional Chinese medicine (TCM), and it has both the characteristics of a “needle” in TCM and a “knife” in Western medicine [15]. Although it has been called feng zhen in ancient literature, acupotomy was reinvented and refined by professor Hanzang Zhu in China in 1976 [16]. Acupotomy is referred to by different names, such as needle-knife, small needle-knife, acupotome, and xiao zhen dao. The mechanism of acupotomy remains unclear and remains to be explored, but acupotomy can be used to release ligaments, joint sacs, and synovium [17]. Some studies have shown that acupotomy therapy can release adhesions, alter the mechanical balance of the knee joint, improve lymphatic circulation, and reduce abnormal tissue pressures [18–21].

Although acupotomy therapy has long been regarded as a key component of the treatment of OA in China and may be considered a safe and promising new treatment for knee OA, the quantitative evidence necessary to estimate its effects is still lacking. Two previous meta-analyses have reported that acupotomy is a safe and effective treatment for knee OA compared to intraarticular sodium hyaluronate and acupuncture [22, 23], but one meta-analysis included only 8 randomized controlled trials (RCTs), and the other meta-analysis included only 6 RCTs. One recent meta-analysis including 12 RCTs [24] attempted to assess the efficacy and safety of acupotomy compared to acupuncture. However, these meta-analyses have methodological flaws, including a lack of up-to-date RCTs data, insufficient sample sizes to make recommendations, and comparisons between acupotomy and only one specific intervention. Thus, these meta-analyses were unable to determine the overall efficacy of acupotomy in the treatment of knee OA. Therefore, we conducted an updated systematic review and meta-analysis to compare the efficacy of acupotomy with that of other treatments in treating patients with knee OA. This study has been registered on PROSPERO (CRD42020161293).

## 2. Methods

### 2.1. Search Strategy

We performed a comprehensive search in PubMed, the Cochrane Library, EMBASE and four Chinese databases (CNKI, Wan Fang, CBMdisc, and VIP) for articles published through June 2020. We included only RCTs that used acupotomy therapy as the main treatment for adults with knee OA. The Chinese and English search terms included acupotomy, acupotomies, acupuncture treatment, acupotomology, acupotome, needle-knife, needle scalpel, stiletto needle, sword-like needle, miniscalpel, small needle-knife, xiao zhen dao, pharmacoacupuncture, knee osteoarthritis, osteoarthritis of knee, osteoarthritis of the knee, pain, randomized controlled trial, and clinical trial.

### 2.2. Eligibility Criteria

Acupotomy was defined as a new type of minimally invasive surgical treatment for knee OA based on the traditional medical theory and modern surgery. We included RCTs that compared acupotomy therapy with acupuncture, electroacupuncture, or standard western treatment in adults with knee OA. Trials were eligible if the intervention included at least 1 acupotomy intervention, more than 20 subjects in each group, and original data. Studies that used the American College of Rheumatology (ACR) diagnostic criteria in 1995 were eligible [25]. We also considered studies that used the Chinese Orthopedic Association (COA) criteria of 2007 or 2018 [26, 27]. To evaluate the independent effects of the acupotomy intervention, we excluded treatment groups that received other major treatments, and we also excluded reviews, theoretical studies, case reports, and animal studies. There were no language restrictions in the literature search.

### 2.3. Study Selection

Two authors (QLC and RZZ) independently screened all the potentially eligible studies. The titles and abstracts were first screened to exclude irrelevant citations. The full texts of all the articles with potentially relevant abstracts were retrieved and screened according to the study eligibility criteria. Disagreements were resolved by consensus or discussion with a third author (YZ).

Pain intensity was measured using the visual analogue scale (VAS) or the Western Ontario and McMaster Universities Osteoarthritis Index (WOMAC). The VAS pain score and WOMAC pain score were the prespecified primary outcomes in this study. The total effectiveness rate was used to assess overall pain, physical performance, and wellness. The total effectiveness rate (%) was defined as the quotient of the number of patients who were clinically cured, exhibited significant improvement, or exhibited improvement divided by the total number of patients. The total effectiveness rate was assessed based on the number of patients in each of the following categories: “clinically cured” (the pain and swelling in the joints had disappeared, and the active functional state had returned to normal); “significant improvement” (the pain and swelling in the joints were alleviated, and the active functional state had improved significantly); “improvement” (the pain and swelling in the joints were partially alleviated, and the active functional state had improved); and “not cured” (the pain and swelling in the joints remained unchanged, and there was no improvement in active function) [28]. The modified Japanese Orthopedic Association (JOA) activities of the daily living (ADL) score was used to assess pain when walking and pain when going up and down stairs. Lysholm's score was used to assess overall pain and joint function. The total effectiveness rate, JOA score, and Lysholm's score were also measured.

### 2.4. Data Extraction

One author (RZZ) extracted data from the selected studies using a predesigned data extraction table, which included publication information, the origin of study, the study setting, the time frame of the study, patient age, patient sex, the author's definition of knee OA, detailed information on the interventions and controls, outcome measures, a summary of the results, the main conclusion, and adverse reactions (Table 1). The accuracy of the data extracted was verified by another author (ZPX).

### 2.5. Quality Assessment

Study quality was assessed in RevMan V5.3 (the Nordic Cochrane Centre, Cochrane Collaboration) using the Cochrane risk of bias tool [29]. The risk of bias for each of the following domains was assessed for each study: (1) random sequence generation, (2) allocation concealment, (3) blinding of the participants and personnel, (4) blinding of the outcome assessments, (5) incomplete outcome data, (6) selective reporting, and (7) other bias. Each study included was rated as having a high, low, or unclear risk of bias. Two authors (YZ and MES) evaluated all the data extracted and quality ratings for consistency and resolved disagreements. Disagreements were resolved by discussion with a third author (RTW).

### 2.6. Data Synthesis and Statistical Analysis

We qualitatively synthesized all the included studies (Table 1). The included studies on pain were synthesized based on the VAS pain score and the WOMAC pain score separately. The VAS score ranged from 0 points (no pain) to 10 points (worst possible pain). The WOMAC pain score ranged from 0 points to 20 points, with a lower score representing a better outcome. Lysholm's score ranged from 0 points to 100 points, and the modified JOA score ranged from 0 points to 55 points, with a higher score representing a better outcome.

All analyses were conducted using RevMan V5.3. For the meta-analysis of the VAS pain score, WOMAC pain score, JOA score, and Lysholm's score, we combined studies using the mean difference (MD); a positive MD indicated that the effect of acupotomy therapy was favourable compared with the control therapy. For the total effectiveness rate, we combined studies using the risk ratio (RR) in the meta-analysis, and an RR of the total effectiveness rate greater than 1 indicated that acupotomy was more effective than was the control therapy. We evaluated heterogeneity using the *I*^2^ statistic. *p* values < 0.05 were considered to indicate statistical significance in all the results.

## 3. Results

### 3.1. Results of the Literature Search and Selection Processes

**Figure 1:**
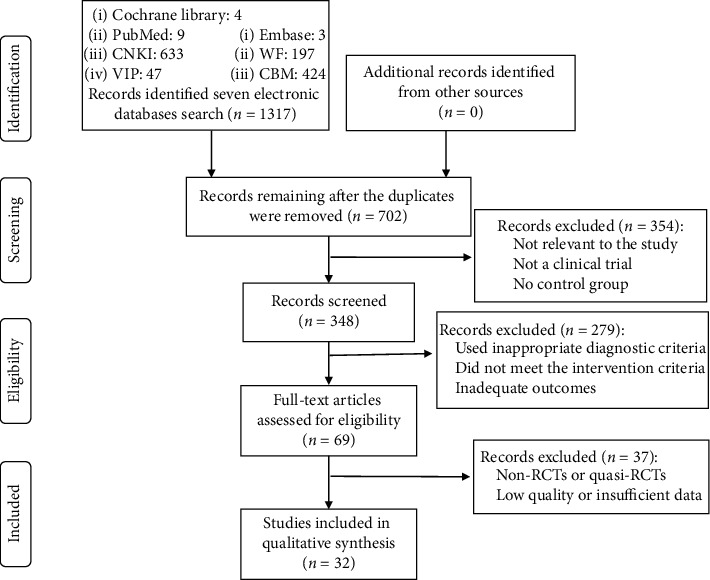
Study selection flow chart.

We screened a total of 1317 studies that were retrieved from 3 English databases and 4 Chinese databases. After initially screening 348 potentially relevant abstracts, we excluded 279 because they did not meet the inclusion criteria. Thirty-seven articles were excluded due to lack of randomization or the absence of a control group and insufficient data for the meta-analysis. Finally, 32 RCTs [19, 30–60], which included 3021 patients (62.96% female) and were published between 2007 and 2020, met our inclusion criteria. The details of the study selection process are summarized in Figure 1.

### 3.2. Included Studies

Table 1 describes the studies and patient characteristics of the included studies. All 32 RCTs [19, 30–60] were conducted in China, and the total sample size of the included RCTs ranged from 41 to 324 (median: 74). The mean age ranged from 47 to 66 years (median: 57 years), and the percentage of females ranged from 42.57% to 87.5% (median: 60%). The disease duration ranged from 4 to 152 months (median: 33 months).

The typical acupotomy therapy involved releasing soft tissue adhesions and was performed once a week for 1–5 weeks until the pain was relieved. Additional massage therapy after acupotomy was included in 2 studies, and functional training was included in 1 study. The control group treatments included acupuncture (8 studies), electroacupuncture (10 studies), sodium hyaluronate (8 studies), radiofrequency electrotherapy (1 study), and NSAIDs (5 studies). The NSAIDs used included oral NSAIDs (3 celecoxib and 1 diclofenac sodium) and topical NSAIDs (1 votalin emulsion). The treatment duration ranged from 1 to 5 weeks (median: 3 weeks).

**Figure 2:**
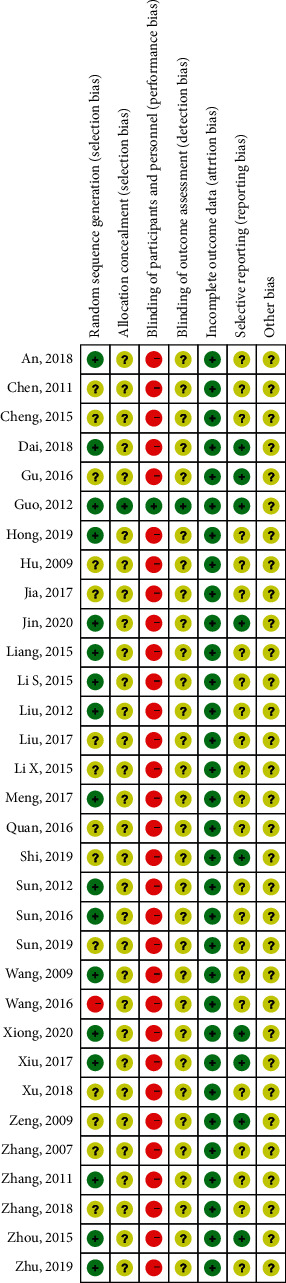
Risk of bias summary.

**Figure 3:**
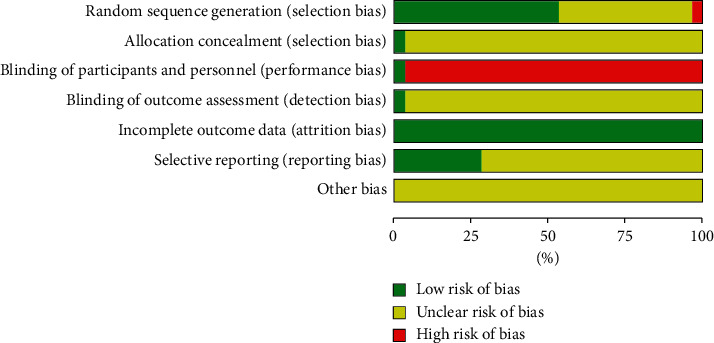
Risk of bias distribution graph.

The quality (risk of bias) of the trials was assessed using the Cochrane Collaboration tool, with modifications.  Figure 2 describes the study quality, and Figure 3 describes the overall risk of bias distribution among the studies included. The overall bias quality for the trials was modest. The randomization process was adequate in 17 trials (53.13%), unclear in 14 trials (43.75%), and indicated a high risk of bias in 1 trial (3.13%). One trial (3.13%) reported appropriate allocation concealment methods, but 31 trials (96.88%) were at high risk of bias. Blinding of the participants and personnel occurred in 1 trial (3.13%), but 31 trials were considered to have a high risk of bias (96.88%). Blinding of the outcomes occurred in 1 trial (3.13%), but whether blinding was performed was unclear in 31 trials (96.88%). All studies reported the similarity of the study groups at baseline (100%).

### 3.3. Meta-Analysis

Among the thirty-two eligible RCTs, twenty-two trials [30–34, 36, 37, 39, 40, 42–47, 49, 50, 53, 55, 58–60] reported the VAS pain score for the individuals who underwent acupotomy therapy and controls. Nine trials [19, 35, 38, 41, 46, 48, 50, 52, 60] reported the WOMAC pain score. Furthermore, twenty-three trials [19, 30–42, 44–46, 48, 52, 55, 58–60] evaluated overall pain, physical performance, and wellness using the total effectiveness rate. Three trials [30, 37, 55] used Lysholm's score and five trials [42, 51, 54, 56, 57] used the JOA score to evaluate overall pain and function.

#### 3.3.1. VAS Pain Score

**Figure 4:**
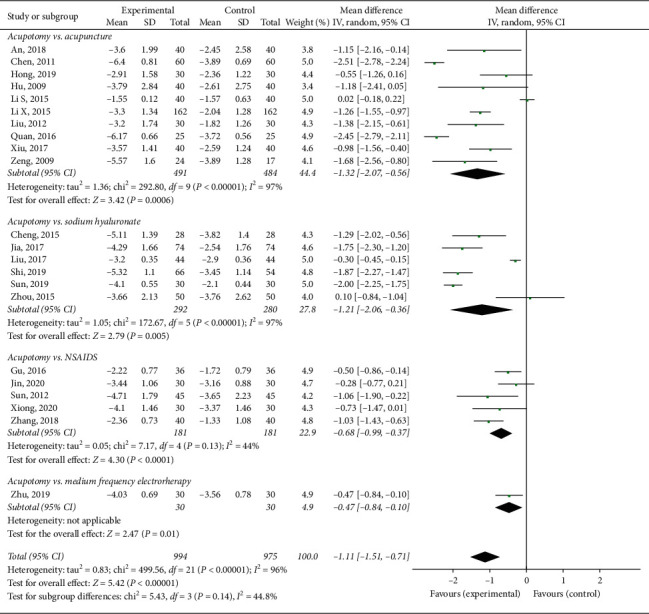
Effect of acupotomy therapy on the VAS pain score.

Twenty-two trials [30–34, 36, 37, 39, 40, 42–47, 49, 50, 53, 55, 58–60] involving 1969 patients were included in the meta-analysis of pain using the VAS pain score. The results of the random-effects meta-analysis indicated that the patients in the acupotomy groups had significantly lower pain scores than did those in the sodium hyaluronate injection, NSAID, acupuncture, electroacupuncture, and medium frequency electrotherapy control groups (MD = −1.11; 95% CI, −1.51 to −0.71; *p* < 0.00001) after 1–5 weeks of treatment. The level of heterogeneity (*I*^2^) in the VAS score was 96% (Figure 4).

The subgroup analysis exploring the improvement in the VAS pain score among different control groups showed that acupotomy therapy has a larger effect than does acupuncture or electroacupuncture (MD = group analysis exploring the impro 0.0006), intraarticular sodium hyaluronate injection (MD = −1.21; 95% CI, −2.06 to −0.36; *p* = 0.005), NSAIDs (MD = −0.68; 95% CI, −0.99 to −0.37; *p* < 0.0001), and medium frequency electrotherapy (MD = −1.11; 95% CI, −1.51 to −0.71; *p* = 0.01) (Figure 4).

#### 3.3.2. WOMAC Pain Score

**Figure 5:**
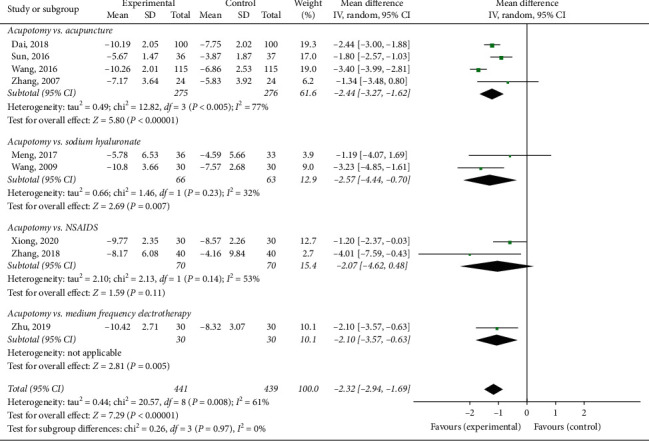
Effect of acupotomy therapy on the WOMAC pain score.

Nine trials [19, 35, 38, 41, 46, 48, 50, 52, 60] involving 880 patients were included in the meta-analysis of pain using the WOMAC pain score. The results of the random-effects meta-analysis indicated that the patients in the acupotomy groups had significantly lower pain scores than did those in the sodium hyaluronate, celecoxib, acupuncture, and electrotherapy control groups (MD = −2.32; 95% CI, −2.94 to −1.69; *p* < 0.00001) after 1–5 weeks of treatment. The level of heterogeneity (*I*^2^) of the WOMAC pain score was 61% (Figure 5).

The subgroup analysis exploring the improvement in the WOMAC pain score among different control groups showed that acupotomy therapy had a larger effect than did acupuncture or electroacupuncture (MD = −2.44; 95% CI, −3.27 to −1.62; *p* < 0.00001), intraarticular sodium hyaluronate injection (MD = −2.57; 95% CI, −4.44 to −0.70; *p* = 0.007), NSAIDs (MD = −2.07; 95% CI, −4.62 to −0.48; *p* = 0.11), and medium frequency electrotherapy (MD = −2.10; 95% CI, −3.57 to −0.63; *p* = 0.005) (Figure 5).

#### 3.3.3. The Total Effectiveness Rate

**Figure 6:**
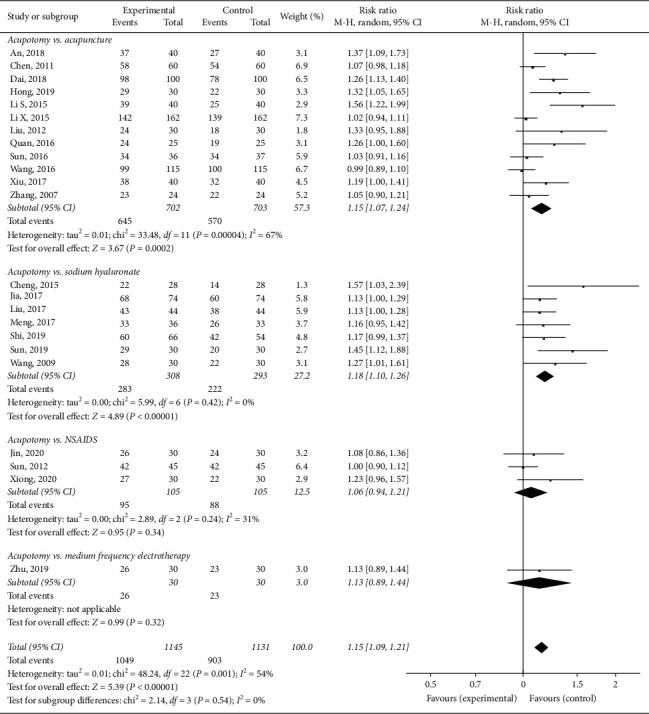
Effect of acupotomy therapy on the total effectiveness rate.

Twenty-three trials [19, 30–42, 44–46, 48, 52, 55, 58–60] involving 2276 patients were included in the meta-analysis of the total effectiveness rate of acupotomy compared to those of acupuncture, electroacupuncture, diclofenac sodium, intraarticular hyaluronate injection, and electrotherapy. The results from our meta-analysis with a random-effects model showed that acupotomy improved the clinical effectiveness rate by 15% (RR = 1.15; 95% CI, 1.09–1.21; *p* < 0.00001), with a moderate degree of heterogeneity (*I*^2^ = 54%). Our meta-analysis showed that 2–5 weeks of acupotomy can improve clinical symptoms such as overall pain, physical performance, and wellness in patients with knee OA (Figure 6).

The subgroup analysis exploring the improvement in the total effectiveness rate among different control groups showed that acupotomy therapy had a larger effect than did acupuncture or electroacupuncture (RR = 1.15; 95% CI, 1.07–1.24; *p* = 0.0002), intraarticular sodium hyaluronate injection (RR = 1.18; 95% CI, 1.10–1.26; *p* < 0.00001), NSAIDs (RR = 1.06; 95% CI, 0.94–1.21; *p* = 0.34), and medium frequency electrotherapy (RR = 1.13; 95% CI, 0.89–1.44; *p* = 0.32) (Figure 6).

#### 3.3.4. Lysholm's Score

**Figure 7:**
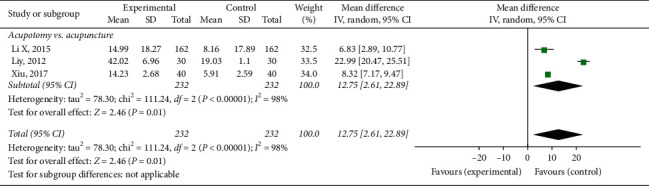
Effect of acupotomy therapy on Lysholm's score.

Three trials [30, 37, 55] involving 464 patients were included in the meta-analysis of the joint function outcomes using Lysholm's score. The results of the random-effects meta-analysis indicated that the patients in the acupotomy groups had significantly better joint function than did those in the acupuncture control groups (MD = 12.75; 95% CI, 2.61–22.89; *p* = 0.01) after 2–4 weeks of treatment. The level of heterogeneity (*I*^2^) in Lysholm's score was 98% (Figure 7).

#### 3.3.5. JOA Score

**Figure 8:**
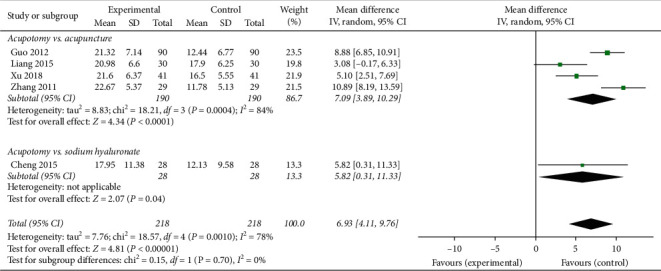
Effect of acupotomy therapy on the JOA score.

Five trials [42, 51, 54, 56, 57] involving 436 patients were included in the meta-analysis of the pain outcomes using the JOA score. The results of the random-effects meta-analysis indicated that the patients in the acupotomy groups had significantly lower pain scores than did those in the sodium hyaluronate and acupuncture control groups (MD = 6.39; 95% CI, 4.11–9.76; *p* < 0.00001) after 3–5 weeks of treatment. The level of heterogeneity (*I*^2^) in the JOA score was 78% (Figure 8).

The subgroup analysis exploring the improvement in the JOA score among different control groups showed that acupotomy therapy had a larger effect than did acupuncture or electroacupuncture (MD = 7.09; 95% CI, 3.89–10.29; *p* < 0.0001) and intraarticular sodium hyaluronate injection (RR = 5.82; 95% CI, 0.31–11.33; *p* = 0.04) (Figure 8).

## 4. Discussion

This systematic review and meta-analysis of 32 RCTs including 3021 individuals indicated that acupotomy therapy has larger beneficial effects than do standard Western medication, Chinese acupuncture, and electroacupuncture for knee OA. In addition, many studies have shown that acupuncture and electroacupuncture are beneficial for knee OA in alleviating pain and improving physical function [61–64]. Overall, acupotomy therapy appears to be a safe method for alleviating pain in people with knee OA.

Our findings are supported by the existing evidence. Zhao et al. [65] reported that according to 7 trials using acupotomy combined with sodium hyaluronate for 5 weeks, this combination therapy is more effective than sodium hyaluronate alone in treating knee OA. Another review of 12 RCTs by Fu et al. [66] suggested that acupotomy combined with ozone appears to have some advantages for treating knee OA. One study [67] reported that the combination of acupotomy therapy and an intraarticular injection showed better short-term and long-term effects than did the intraarticular injection only (control group). Furthermore, Cheng et al. [68] used acupotomy therapy and the bleeding method for knee OA patients, and the total effective rate reached 96.7%. The results of these reviews and studies agreed with our findings and indicated that acupotomy therapy is beneficial in reducing pain and improving the physical function of individuals with knee OA. However, acupotomy was combined with other treatments in these reviews; so, it is hard to determine the exact effects of acupotomy alone. Our study included only RCTs that compared acupotomy with acupuncture, electroacupuncture, sodium hyaluronate, NSAIDs, or other treatments, so the effect of acupotomy on knee OA was clearer.

Despite the lack of knowledge about the biologic mechanisms of acupotomy therapy, it is likely to relieve contractures in muscles and fasciae, relieve pain, and improve the function of the knee joint by releasing adhesive tissue [69]. The growing body of evidence is beginning to shed light on the potential mechanisms by which acupotomy therapy relieves the symptoms of knee OA. One study [70] showed that acupotomy can significantly reduce the magnitude of knee effusion and synovial thickness in patients with knee OA, as assessed by musculoskeletal ultrasound. One study [71] indicated that the centre of gravity is closer to the original point, and weight bearing is improved after acupotomy treatment. One animal study [72] showed that acupotomy can significantly change the behaviour and morphology and significantly improve the mechanical properties of the quadriceps femoris tendon. Recent studies [73–75] have suggested that acupotomy therapy can promote the repair of cartilage cells by activating the FAK-PI3K signalling pathway, promote cartilage cell metabolism, and regulate the PERK-eIF2*α*-CHOP signalling pathway. Several studies [76, 77] have already shown an association between increases in the expression levels of the integrin *β*1, col-II, and aggrecan proteins and decreases in the expression of BAX, caspase-3, and MMP-3 proteins. In addition, acupotomy may also have an anti-inflammatory effect by suppressing the expression of inflammatory cytokines such as interleukin (IL)-1*β*, IL-6, and TNF-*α* [20]. Overall, the mechanisms by which acupotomy relieves the symptoms of knee OA are still not clear, and there is accumulating evidence suggesting that acupotomy alters biomechanics, inhibits chondrocyte apoptosis, reduces inflammatory factors and anti-inflammation, inhibits pain signal transduction, and alleviates pain [78–80].

Our study has limitations. First, the overall methodological quality of the RCTs was moderate. Many of the included RCTs had a high risk of bias. Only one study reported double blinding and allocation concealment, and there were no placebo-controlled studies. Second, these studies were short-term, and their treatment did not exceed 6 weeks; therefore, a longer duration of follow-up is needed in future research. Third, the reporting of adverse events was insufficient. Only 1 trial [43] reported three cases of redness and swelling in a control group treated with sodium hyaluronate, and one trial [46] reported 3 cases of subcutaneous bruising after the acupotomy intervention and 2 cases of a stomach ache in the control group receiving a celecoxib capsule. Three trials reported no adverse events, but 27 trials did not mention adverse events. Acupotomy therapy appears to be a safe method with no severe side effects, but it is important to assess adverse events in future studies. Fourth, despite the statistically significant and beneficial effects of acupotomy on pain and function in patients with knee OA, the clinically important benefits of acupotomy remain to be determined. Many challenges remain, and the potential benefits of acupotomy therapy for knee OA need to be further evaluated through clinical trials that employ more rigorous methodologies.

## 5. Conclusions

In summary, acupotomy therapy may be effective in reducing pain and improving the physical function of individuals with knee OA. Despite the limited quality of the trials included in this review, our study provides new and valuable information. Chinese acupotomy therapy may be effective in treating knee OA. More rigorous randomized controlled trial designs are needed in the future.

## Figures and Tables

**Table 1:** Characteristics of the 32 included studies on Chinese acupotomy therapy for knee OA.

Author (y)	Diagnostic criteria	N (female, %）	Age (%)	Disease duration (months)	Duration (wks)	Acupotomy therapy	Controls	Main outcomes	Results (treatment vs. control)	*p* value
Li X. (2015) [30]	COA criteria (2007)	324 (57.41%)	64	61	3	Release soft tissue adhesion, once/wk, 3 times	Acupuncture, 30 min, 6 times/wk, 3 wks	VAS pain Lysholm's score Total effectiveness rate	2.83 vs. 3.94 89.55 vs. 80.64 87.65 vs. 85.80	<0.05 <0.05 < 0.05

Li S. (2015) [31]	ACR knee OA criteria (1995)	67 (43.28%)	55	Not mentioned	2	Release soft tissue adhesion, once/wk, 2 times	Electroacupuncture, 20 min, once/day, 10 times	VAS pain Total effectiveness rate	1.58 vs. 2.69 97.5 vs. 62.5	<0.05 <0.01

Sun (2019) [32]	Not mentioned	60 (71.67%)	59	152	4	Release soft tissue adhesion, once/wk, 4 times	Sodium hyaluronate, 2 ml, once/wk, 4 wks	VAS pain Total effectiveness rate	3.41 vs. 5.48, 96.67 vs. 66.67	<0.01 <0.05

Shi (2019) [33]	COA criteria (2018)	120 (67.5%)	58	21	4	Release soft tissue adhesion, once/wk, 4 times	Sodium hyaluronate, 2.5 mg, once/6 days, 5 times	VAS pain Total effectiveness rate	3.01 vs. 4.81, 90.91 vs. 77.78	<0.05 <0.05

Hong (2019) [34]	COA criteria (2007)	61 (67.21%)	58	15	3	Release soft tissue adhesion, once/wk, 3 times	Acupuncture, 5 times/wk, 3 wks	VAS pain Total effectiveness rate	1.81 vs. 2.7 96.7 vs. 73.3	<0.05 <0.05

Wang (2016) [35]	COA criteria (2007)	230 (63.91%)	52	14	2	Release soft tissue adhesion, once	Acupuncture, 20 min, 1-2 times/wk, 2 wks	WOMAC pain Total effectiveness rate	86.09 vs. 86.96	<0.05 <0.05

Jin (2020) [36]	ACR knee OA criteria	60 (58.33%)	57	89	4	Release soft tissue adhesion, twice/wk, 8 times	Celecoxib capsules, 200 mg, twice/day, 4 wks	VAS pain Total effectiveness rate	3.23 vs. 3.67 86.7 vs. 80	<0.05 <0.05

Xiu (2017) [37]	COA criteria (2007)	80 (56.25%)	53	73	2	Release soft tissue, adhesion, once/wk, 4 times; Massage therapy, once/2 days, 14 times	Acupuncture, 30 min, once every other day, 7 times; massage therapy, once/2 days, 14 times	VAS pain Lysholm's score Total effectiveness rate	2.51 vs. 3.52 58.51 vs. 51.02 95 vs. 80	<0.05 <0.01 <0.01

Dai (2018) [38]	COA criteria (2007)	200 (58.5%)	53	17	2	Release soft tissue adhesion, once	Acupuncture, 20 min, 1-2 times/wk, 2 wks	WOMAC Pain Total effectiveness rate	8.52 vs. 10.81 98 vs. 78	<0.05 < 0.01

Jia (2017) [39]	COA criteria (2007)	148 (42.57%)	59	77	5	Release soft tissue adhesion, once/wk, 5 times	Sodium hyaluronate, 25 mg, once/wk, 5 times	VAS pain Total effectiveness rate	2.19 vs. 3.88 91.89 vs. 81.08	<0.05 <0.05

Quan (2016) [40]	COA criteria (2007)	50 (72%)	56	78	2	Release soft tissue adhesion, once/wk, 2 times	Electroacupuncture, 30 min, once/2 days, 7 times	VAS pain Total effectiveness rate	1.28 vs. 3.71 96 vs. 76	<0.01 <0.05

Sun (2016) [41]	COA criteria (2007)	73 (71.23%)	56	11	4	Release soft tissue adhesion, once/wk, 4 times	Acupuncture, 30 min, 5 times/wk, 4 wks	WOMAC Pain Total effectiveness rate	3.95 vs. 7.46 94.44 vs. 91.89	<0.05 <0.05

Cheng (2015) [42]	COA criteria (2007)	56 (44.64%)	57	32	5	Release soft tissue adhesion, once/wk, 4 times	Sodium hyaluronate, 2 ml, once/wk, 5 wks	VAS pain JOA assessment Total effectiveness rate	2.15 vs. 3.52 46.37 vs. 39.81 78.57 vs. 50	<0.05 <0.05 <0.05

Zhou (2015) [43]	COA criteria (2007)	100 (64%)	58	12	4	Release soft tissue adhesion, once/wk, 4 times	Sodium hyaluronate, 2 ml, once/wk, 4 wks	VAS pain	1.52 vs. 1.49	>0.05

Sun (2012) [44]	COA criteria (2007)	90 (73.33%)	59	13	4	Release soft tissue adhesion, once/wk, 1–4 times, until pain relieves	Diclofenac sodium, 75 mg, once/day, 20 days, oral pill	VAS pain Total effectiveness rate	2.76 vs. 3.84 93.33 vs. 93.33	<0.05 <0.05

Chen (2011) [45]	ACR knee OA criteria (1995)	120 (69.17%)	60	45	2	Release soft tissue adhesion, once/wk, 2 times; Massage therapy, once/2 days, 14 times	Electroacupuncture, 30 min, once every other day, 7 times; massage therapy, once/2 days, 14 times	VAS pain Total effectiveness rate	1.27 vs. 3.64 96.7 vs. 90	<0.01 <0.05

Xiong (2020) [46]	COA criteria (2007)	60 (56.67%)	54	69	3	Release soft tissue adhesion, once/wk, 3 times	Celecoxib capsules, 200 mg, once/day, 3 wks	VAS pain WOMAC pain Total effectiveness rate	5.77 vs. 4.57 11.4 vs. 9.17 90 vs. 73.33	<0.05 <0.05 <0.05

Hu (2009) [47]	ACR knee OA criteria (1995)	80 (50%)	66	67	4	Release soft tissue adhesion, once/wk, 4 times	Electroacupuncture, 30 min, once/day, 4 wks	VAS pain	3.52 vs. 5.26	<0.05

Wang (2009) [48]	ACR knee OA criteria (1995)	60 (66.67%)	49	61	5	Release soft tissue adhesion, 1 time	Sodium hyaluronate, 2 ml, once/wk, 5 wks	WOMAC pain Total effectiveness rate	1.8 vs. 5.33 93.33 vs. 73.33	<0.01 <0.05

Zeng (2009) [49]	ACR knee OA criteria (1995)	41 (68.29%)	Not mentioned	Not mentioned	3	Release soft tissue adhesion, once/wk, 1–3 times, until pain relieves	Electroacupuncture, 30 min, 3 times/wk, 3 wks	VAS pain	1.49 vs. 2.85	<0.01

Zhang (2018) [50]	COA criteria (2007)	80 (60%)	58	59	1	Release soft tissue adhesion, 2 times	Celecoxib, 400 mg, once/day, 1 wk Omeprazole, 20 mg, once/day, 1 wk	VAS pain WOMAC pain	2.54 vs. 3.56 7.3 vs. 8.93	<0.05 <0.05

Xu (2018) [51]	ACR knee OA criteria	82 (56.10%)	58	34	3	Release soft tissue adhesion, once/wk, 1–3 times	Electroacupuncture, 3 times/wk, 10 times	JOA assessment	48.9 vs. 43.2	<0.05

Meng (2017) [52]	COA criteria (2007)	69 (44.93%)	56	54	4	Release soft tissue adhesion, once/wk, 4 times	Sodium hyaluronate, 2 ml, once/wk, 4 wks	WOMAC pain Total effectiveness rate	15.36 vs. 17.55 91.67 vs. 78.79	<0.05 <0.05

Gu (2016) [53]	ACR knee OA criteria (1995)	75 (69.33%)	57	28	2	Release soft tissue adhesion, once/wk, 2 times	Votalin emulsion, 3 times/day, 2 wks	VAS pain	2.06 vs. 2.64	<0.05

Liang (2015) [54]	ACR knee OA criteria (1995)	60 (73.33%)	59	10	3	Release soft tissue adhesion, once/wk, 1–3 times; functional training, 3 wks	Electroacupuncture, 3 times/wk, 10 times; functional training, 3 wks	JOA assessment	48.26 vs. 43.94	<0.05

Liu (2012) [55]	COA criteria (2007)	60 (51.67%)	63	38	4	Release soft tissue adhesion, once/wk, 1 month	Acupuncture and moxibustion, once/day, 1 month	VAS pain Lysholm's score Total effectiveness rate	2.01 vs. 3.32 23.3 vs. 48.35 80 vs. 60	<0.05 <0.05 <0.05

Guo (2012) [56]	ACR knee OA criteria (1995)	180 (65.56%)	60	62	3	Release soft tissue adhesion, once/wk, 3 wks	Electroacupuncture, 3 times/wk, 3 wks	JOA assessment	43.66 vs. 39.27	<0.01

Zhang (2011) [57]	ACR knee OA criteria (1995)	58 (53.45%)	53	20	3	Release soft tissue adhesion, once/wk, 1–3 times, until pain relieves	Electroacupuncture, 3 times/wk, 3 wks	JOA assessment	41.33 vs. 31.79	<0.01

Zhang (2007) [19]	ACR knee OA criteria (1995)	48 (87.50%)	61	31	3	Release soft tissue adhesion, once/wk, 1–3 times, until pain relieves	Electroacupuncture, twice/wk, 3 wks	WOMAC pain Total effectiveness rate	3 vs. 4.13 95.8 vs. 91.7	<0.01 >0.05

Liu (2017) [58]	Not mentioned	88 (54.55%)	63	77	5	Release soft tissue adhesion, once/wk, 4 times	Sodium hyaluronate 20–30 mg, once/wk, 5 times	VAS pain Total effectiveness rate	1.5 vs. 1.9 97.7 vs. 88.4	<0.05 <0.05

An (2018) [59]	Not mentioned	80 (53.75%)	47	30	3	Release soft tissue adhesion, once/wk, 3 times	Acupuncture, 20 min, once/wk, 3 wks	VAS pain Total effectiveness rate	2.67 vs. 4.18 92.5 vs. 67.5	<0.05 <0.01

Zhu (2019) [60]	COA criteria (2007)	60 (60%)	53	4	2	Release soft tissue adhesion, once/wk, 2 times	Medium frequency electrotherapy, 6 times/wk, 2 wks	VAS pain WOMAC pain Total effectiveness rate	2.47 vs. 2.78 5.26 vs. 6.39 89.7 vs. 82.9	<0.05 <0.05

^*∗*^ACR, American College of Rheumatology; COA, Chinese Orthopedic Association; y, year; *N,* number of patients included; VAS, visual analogue scale; WOMAC, Western Ontario and McMaster Universities Osteoarthritis Index; JOA, Japanese Orthopedic Association.

(1) VAS pain score: 0–10; lower score = better outcome. (2) WOMAC pain score: 0–20; it was assessed with the following five items: pain during walking, stair climbing, resting, weight bearing, and pain at night. Each subscale used the following descriptors: none (0 points), mild (1 point), moderate (2 points), severe (3 points), and extreme (4 points); lower score = better outcome. (3) The total effectiveness rate (%) was defined as the quotient of the number of patients who were clinically cured, exhibited significant improvement, and exhibited improvement divided by the total number of patients. It assesses overall pain, physical function, and wellness; a higher score = better outcome. (4) Lysholm's score: 0–100, 100 points indicated no symptoms, 80–99 indicated “excellent”, 70–79 indicated “good,” 60–69 indicated “medium”, and less than 60 indicated “poor”; higher score = better outcome. (5) Modified JOA score [57]: 0–55, including pain when walking (30 points) and pain when going up and down stairs (25 points); higher score = better outcome.

## Supplementary Materials

Supplementary Materials Additional file 1: VAS pain score funnel plot; additional file 2: WOMAC pain score funnel plot; additional file 3: the total effectiveness rate funnel plot; additional file 4: Lysholm's score funnel plot; additional file 5: JOA score funnel plot; additional file 6: image of acupotomy therapy, 0.8  mm × 80  mm (Huayou Medical Co., China).
