# Supplementary material for: Acupotomy Therapy for Knee Osteoarthritis Pain: Systematic Review and Meta-Analysis
Source: Evid Based Complement Alternat Med. 2020 Oct 31;2020:2168283. doi: 10.1155/2020/2168283 (PMC7648689; doi:10.1155/2020/2168283)
Supplement: Supplementary Materials — Additional file 1: VAS pain score funnel plot; additional file 2: WOMAC pain score funnel plot; additional file 3: the total effectiveness rate funnel plot; additional file 4: Lysholm's score funnel plot; additional file 5: JOA score funnel plot; additional file 6: image of acupotomy therapy, 0.8 mm × 80 mm (Huayou Medical Co., China). [file 2168283.f1.doc]

**
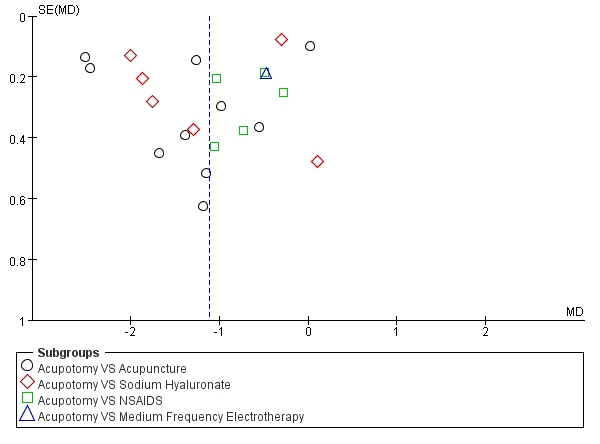
**

**Additional file 1: VAS pain score funnel plot.**

**
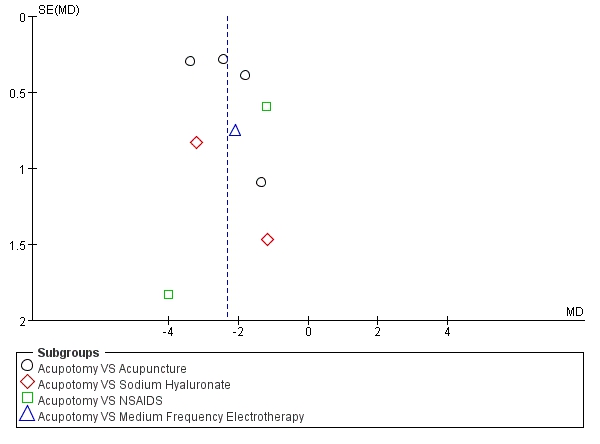
**

**Additional file 2: WOMAC pain score funnel plot.**

**
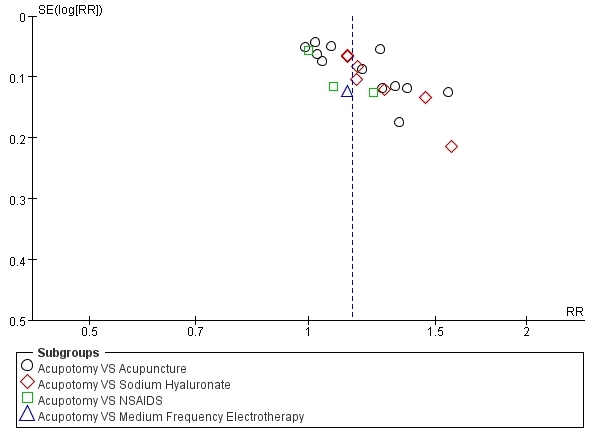
**

**Additional file 3: The total effectiveness rate funnel plot.**

**
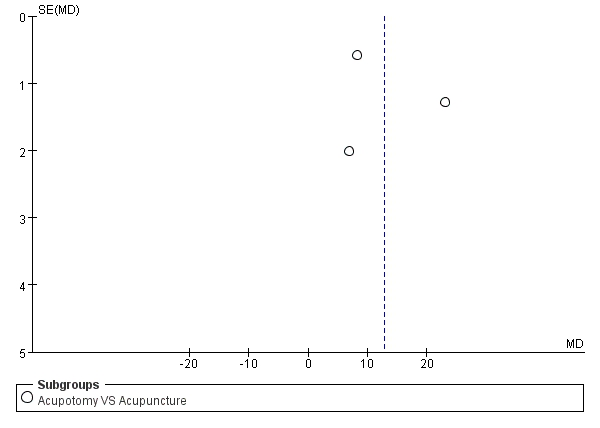
Additional file 4: Lysholm’s score funnel plot.
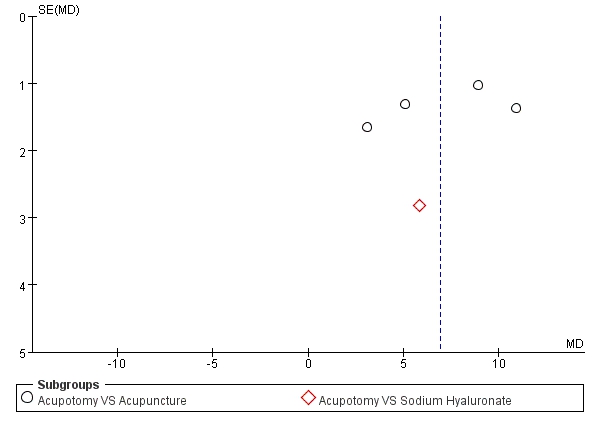
A****dditional file 5: JOA score funnel plot.
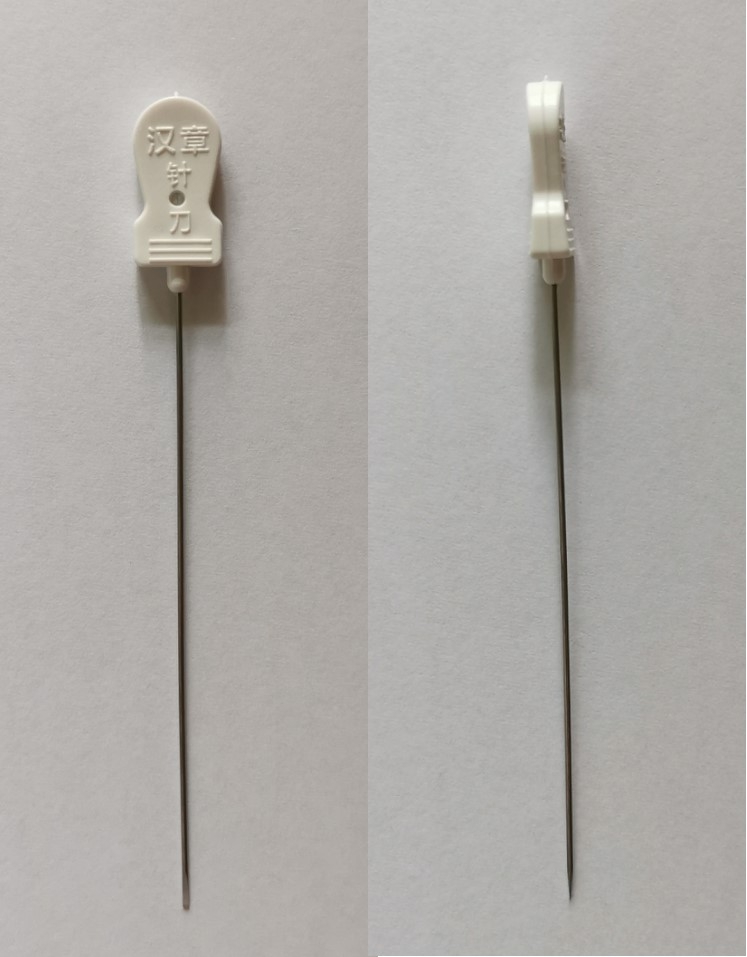
**

**Additional file 6: Image of acupotomy therapy, 0.8mm×80mm (Huayou Medicial Co., China).**
